# Changes in wing resonance in dried preserved crickets

**DOI:** 10.1098/rsos.241147

**Published:** 2024-12-18

**Authors:** Sophia Laskri, Lewis B. Holmes, Thomas Dixon, Tony Robillard, Fernando Montealegre-Z

**Affiliations:** ^1^ School of Life and Environmental Sciences, Green Lane, Lincoln LN6 7DL, UK; ^2^ Institut UniLaSalle, Mont-Saint-Aignan 76130, France; ^3^ Institut de Systématique, Evolution, Biodiversité (ISYEB), Muséum national d'Histoire naturelle, CNRS, SU, EPHE-PSL, UA, CP 50, Paris 75005, France

**Keywords:** Ensifera, wing resonance, carrier frequency, stridulation, desiccation, laser Doppler vibrometry

## Abstract

Male crickets sing to attract females for mating. Sound is produced by tegminal stridulation, where one wing bears a plectrum and the other a wing vein modified with cuticular teeth. The carrier frequency (*f_c_*) of the call is dictated by the wing resonance and the rate of tooth strikes. Therefore, the *f_c_* varies across species due to the size of the vibrating membranes on the wings and/or the speed of tooth strikes. But how well is the resonant frequency (*f_o_*) conserved in dried preserved specimens? This project is designed to investigate the gradual change in cricket wing *f_o_* over time and aims to produce equations that help to predict or recover the original natural frequency of wing vibration in dry-preserved crickets and allies. Using laser Doppler vibrometry, we scanned the wings of living specimens to determine their *f_o_*. The specimens were then preserved, allowing us to continue measuring the wings *f_o_* as they desiccate. We found that after the first week, *f_o_* increases steeply, reaching a plateau and stabilizing for the following months. We go on to propose a model that can be used to recover the original *f_c_* of the wings of preserved Ensifera that use pure tones for communication. Models were corroborated using preserved specimens previously recorded and mounted in dry collections for more than 10 years.

## 1. Introduction

**Figure 1.**
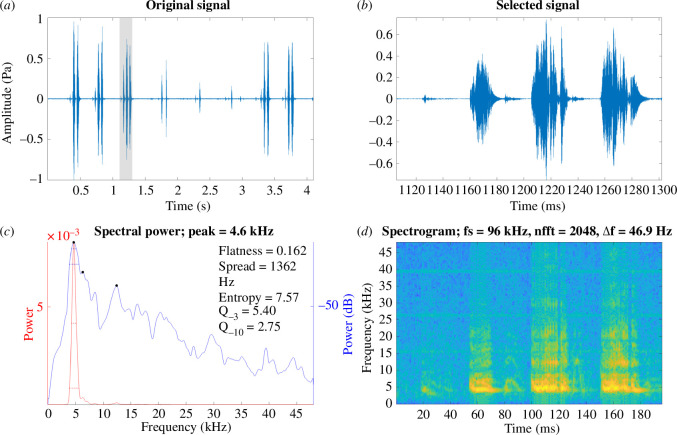
*Gryllus bimaculatus* male calling song under laboratory conditions recorded at a temperature of 19°C. (*a*) A section of the song of *G. bimaculatus*. (*b*) Close-up view of a syllable. (*c*) Power spectrum of a syllable. (*d*) Spectrogram of a syllable where brighter colours indicate greater acoustic power.

Male crickets (Orthoptera: Ensifera: Gryllidae) call over long distances using tegmino-tegminal stridulation, with the main aim of attracting a female partner [1,2]. This song is performed using a plectrum on the anal edge of the wing to pass over a file (a modified vein with cuticular teeth) on the ventral surface of the other wing [3–5]. Cricket wings are at first glance mirror images of each other and could theoretically work in both directions; however, this is not the case, with field crickets generally having a right over left wing arrangement [6,7]. In crickets, the plectrum-tooth strike rate has been shown to equal the resonant frequency (*f_o_*) of the wings and the carrier frequency (*f_c_*) of the animal’s calling songs [7–10]. The wings have two areas primarily associated with their calling songs: a large triangular-shaped harp and a smaller mirror located distally above it (see fig. 1 and table 1 in [7]). The harp is responsible for producing the main amplitude or power component and dominant frequency of the calling song [4,7,11,12]. The mirror generally offers a higher independent *f_o_* than that of the harp, but with much lower amplitude [11,13,14]. In addition, the two fore wings exhibit a slight frequency disparity between them, with the left wing displaying a lower natural *f_o_* than the right wing [15,16]. However, when both wings are engaged together during stridulation, this difference, as well as the mirrors’ high resonances, disappear to produce the calling song [4].

The resonant frequency of a wing is its natural frequency of vibration; this allows the wing to amplify sound waves that match its frequency of vibration [7,11]. Wing resonances have been measured in different ways, from measuring the displacement of talcum powder particles [17] and lycopodium powder [7] under a microscope after sound or mechanical stimulation, measuring near-field sound using probe microphones [7], and recently less invasive methods using contactless laser Doppler vibrometry (LDV) and sympathetic acoustic stimulation [10,14].

If the wings dry in preserved animals, the *f_o_* probably increases due to a change in the stiffness in the wings [18,19], but what precisely happens to the wing resonance in preserved pinned museum specimens has never been investigated. It is currently unknown as to whether dried museum specimens can be used to obtain accurate data regarding the animals’ wing resonance and through that, the *f_c_*. Being able to recover the acoustic properties of dried preserved wings could benefit the field greatly and open new research directions using museum insect collections. This project is intended to explore the change in resonance over time as the sample dries; rigidity plays a major role in the membrane vibratory behaviour. This analysis should provide a valuable insight into the real behaviours and sound production mechanisms of species that are not available as living specimens and potentially even extinct species existing only in museum collections. This could have a positive impact on studies involved in biodiversity and species diversification using sound, helping to bridge the gaps and reveal previously unknown acoustic features of extinct species.

Here, we test the hypothesis that the wing resonance will increase as the specimen dries out due to a change in stiffness and predict that such an increment should reach a limit where the *f_o_* does not change any more.

## 2. Material and methods

### 2.1. Specimens

Specimens from five different species were used for this study: *Macrobinthus jharnae* Bhowmik 1981 (*n* = 3), *Microbinthus pintaudi* Robillard & Dong 2016 (*n* = 3), *Gryllus bimaculatus* De Geer 1773 (*n* = 7), *G. assimilis* Fabricius 1775 (*n* = 5), and *Teleogryllus oceanicus* Le Guillou 1841 (*n* = 5). *Macrobinthus jharnae* and *Mi. pintaudi* specimens used in this study were obtained from maintained colonies at the Muséum national d’Histoire naturelle. Specimens of *G. bimaculatus* (*n* = 20) and *G. assimilis* (*n* = 15) were acquired from laboratory colonies in the Universities of Cambridge and Lincoln, while a small number of *T. oceanicus* (*n* = 8) were obtained from laboratory colonies in the University of St Andrews. All specimens were kept separately in plastic vivaria, fed ad libitum on a mixture of dry cat food and fresh fruit, given a base of kitchen towel space to maintain humidity, with egg boxes as a hiding place. Animals were maintained at 25°C and misted three times a week.

### 2.2. Laboratory recordings

To obtain the carrier frequencies, in the laboratory, the calling songs from males of each species were recorded using a 1/8″ microphone (Brüel & Kjær, Nærum, Denmark) coupled with a Brüel & Kjær 2633 preamplifier (Brüel & Kjær, Nærum, Denmark) and connected to a G.R.A.S. 12AA 2-channel power module (GRAS sound and vibration, Denmark). The microphone’s sensitivity was calibrated with a sound level calibrator (Brüel & Kjær 4231) and the interface of the Polytec Scanning Vibrometer (PSV) software (v. 10.0; Polytec, Waldbronn, Germany). The power module output was connected to PSV acquisition software (Polytec GmbH, Waldbronn, Germany), which used a National Instruments (NI) acquisition board (PCI-4451; National Instruments, Austin, TX, USA). A high-pass filter was set at 1 kHz in order to remove any low-frequency background noise that may interfere with the recordings, with a sample frequency of 100 k samples s^−1^. Calls were recorded at a temperature of 19°C. Males were placed in a wire mesh cylinder suspended 20 cm away from the recording microphone; they were provided with food and water for the duration of the recordings. Each male was recorded up to eight times for a maximum duration of 8 s. The best recordings from each male were selected for analysis. Acoustic analysis of the calls was done using MATLAB 2022 (MathsWorks, Natick, USA).

### 2.3. Recording wing resonance

Forewing resonance was measured using micro-scanning LDV (PSV-500; Polytec GmbH, Waldbronn, Germany). Male specimens of *G. bimaculatus* (*n* = 3), *Ma. jharnae* (*n* = 3) and *Mi. pintaudi* (*n* = 3) were first sedated using chlorethan (Dr. Henning Chloraethyl Spray, Chemische Fabrik, Walldorf GmbH). They were then mounted in a holder with their wings outstretched by putting a small drop of wax made of 50% beeswax (Fisher Scientific, Loughborough, UK) and 50% Colophonium (Sigma-Aldrich Company Ltd, Dorset, UK), in the wing hinge. Acoustic signals for wing excitation consisted of broadband chirps at 2–60 kHz. These were generated through the internal NI card of the Polytec software, amplified (Ultrasonic Power Amplifier, Avisoft Bioacoustics, Glienicke, Germany) and transmitted to a loudspeaker (Ultrasonic Dynamic Speaker Vifa, Avisoft Bioacoustics, Glienicke, Germany) that was positioned 15 cm in front of the tegmina. The reference signal was recorded using the ultrasound-sensitive 1/8″ microphone set-up (Brüel & Kjær, Nærum, Denmark, as described above) positioned in the middle of the two tegmina. This process measures the displacement and velocity values of the wing vibrations during excitation and reveals the frequency at which the wings resonate. Additionally, the tegmina of *G. bimaculatus* (*n* = 4), *G. assimilis* (*n* = 5) and *T. oceanicus* (*n* = 5) were removed and mounted to the tip of a wooden toothpick using a wax made of 50% beeswax (Fisher Scientific, Loughborough, UK) and 50% Colophonium (Sigma-Aldrich Company Ltd, Dorset, UK). The same protocol to measure resonance was repeated on the removed wings.

### 2.4. Preservation of recorded specimens

To test the effects of desiccation on the acoustic properties of the wings, the animals were euthanized by placing them in a killing jar containing cotton wool soaked with nail polish remover (Sainsbury’s Supermarkets Ltd, London). After this, specimens were prepared for pinning following standard protocols for entomological collections. The abdomen was emptied, cleaned and filled with cotton wool with a mixture of 50% talcum powder and 50% boric acid. The animals were then arranged and pinned as in entomological collections with their wings extended, as in the first LDV scan, in an entomological Smith’s box with silica gel. LDV scans were repeated on the preserved specimens every 7 days over the next month to monitor the change in *f_o_*. A final scan was taken 30 weeks after the initial scan to extend experiments beyond four weeks and monitor any change in *f_o_*.

For specimens with removed wings, isolated wings were left mounted to the tips of toothpicks in a box with silica gel. The protocol used to record wing resonance was then repeated on a weekly basis for the next month.

### 2.5. Creating and testing the model

Using the scan data from the alive and then preserved specimens, we hoped to use the *f_o_* change in each individual and plot this against the animals recorded *f_c_*. This model can then be used to recover the original *f_c_* of long-dead preserved specimens by inputting the *f_o_* value obtained from LDV. To test the reliability of the model on long-preserved specimens, using LDV, we scanned the mirror of the right tegmen in response to acoustic stimulation, in a number of additional preserved Ensiferan specimens from entomological collections of which the *f_c_* was known from sound recordings. A single male of each *Typophyllum spurioculis*, *T. bolivari*, *Satizabalus huaca*, *Artiotonus artius* and *Ragoniella pulchella* was scanned from the laboratory collection at the University of Lincoln, Sensory Biology Laboratory. Both *Typophylum* specimens have been pinned and displayed with their wings outstretched since 2000. The *f_c_* of *T. spurioculis* is known to be 13.9 ± 2.2 kHz, while the call of *T. bolivari* is reported as 14.1 kHz. The specimens of *Satizabalus sodalis* and *R. pulchella* have been preserved since 1996 with the *f_c_* known to be 19.8 and 27.6 kHz, respectively. *Artiotonus artius* was recorded and pinned in 1995 with a *f_c_* peak at 40.6 kHz. Lastly, the holotype specimen of *Prophalangopsis obscura* was also scanned. The holotype of *P. obscura* is housed at the London Natural History Museum, UK (specimen NHMUK 013806185). Some time between 1898 and 1939, the specimen was remounted with both wings spread, a position that remains to this day. The *f_c_* is known to be around 4.7 kHz from a reconstruction of the call [20].

## 3. Results

### 3.1. Acoustic signals

**Figure 2.**
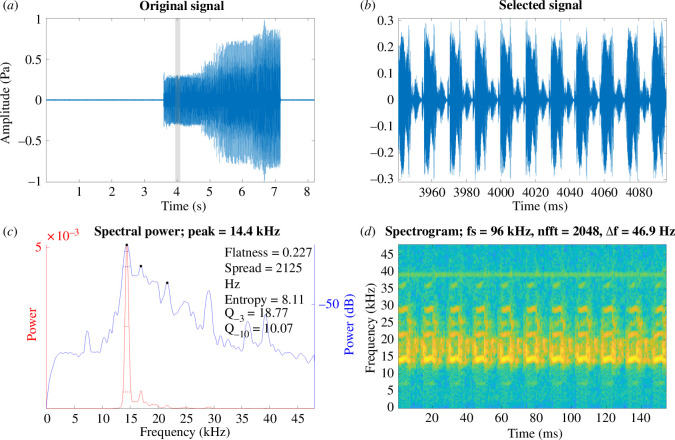
*Macrobinthus jharnae* male calling song under laboratory conditions recorded at a temperature of 19°C . (*a*) A section of the song of *Ma. jharnae*. (*b*) Close-up view of a syllable. (*c*) Power spectrum of a syllable. (*d*) Spectrogram of a syllable where brighter colours indicate greater acoustic power.

**Figure 3.**
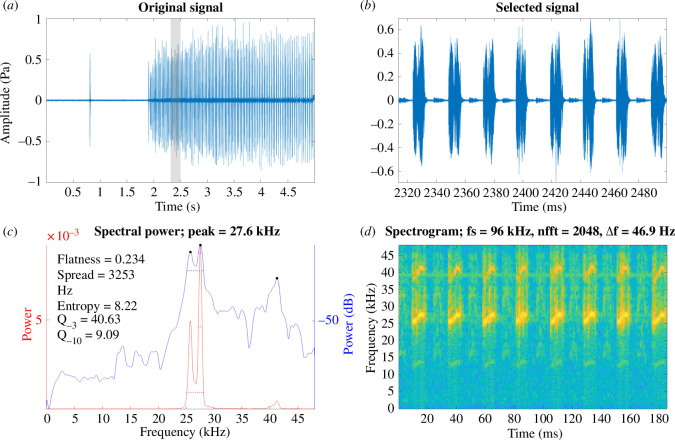
*Microbinthus pintaudi* male calling song under laboratory conditions recorded at a temperature of 19°C. (*a*) A section of the song of *Mi. pintaudi*. (*b*) Close-up view of a syllable. (*c*) Power spectrum of a syllable. (*d*) Spectrogram of a syllable where brighter colours indicate greater acoustic power.

Recordings of the calling song of *G. bimaculatus*, obtained in the laboratory at 19°C, show a *f_c_* of 4.8 ± 0.203 kHz (figure 1*c*; table 1). The *f_c_* of *Ma. jharnae* and *Mi. pintaudi* show energy at much higher frequencies at 15.8 ± 0.314 kHz (figure 2*c*; table 1) and 27 ± 0.417 kHz (figure 3*c*; table 1), respectively. The *f_c_* of *G. assimilis* shows a dominant and fundamental peak at 5.3 ± 0.398 kHz, while *T. oceanicus* was lower at 4.6 ± 0.204 kHz (table 1).

**Table 1.** Changes in wing resonance over time with carrier frequency and initial wing resonance. *f_c_*, carrier frequency; *f_o_*, resonant frequency.

species	wing	*f_c_* (kHz)	*f_o_* week 0 (kHz)	*f_o_* week 1 (kHz)	*f_o_* week 2 (kHz)	*f_o_* week 3 (kHz)	*f_o_* week 4 (kHz)	*f_o_* week 30 (kHz)
*Gryllus bimaculatus* (*n* = 3)	right	4.8	4.72	6.1	5.96	6.03	6.04	6.09
	left		4.49	6.46	6.53	6.51	6.53	6.51
*Macrobinthus jharnae* (*n* = 3)	right	15.88	15.05	17.39	17.2	17.2	17.18	17.34
	left		11.6	14.96	15.1	15.3	15.34	15.72
*Microbinthus pintaudi* (*n* = 3)	right	27	28.46	30.38	30.16	30.51	30.46	29.99
	left		27.64	30.1	30.83	30.8	30.9	31.04
*Gryllus bimaculatus* (wings removed) (*n* = 4)	right	4.8	4.26	5.14	5.21	5.23	5.32	
	left		4.55	5.41	5.73	5.69	5.65	
*Gryllus assimilis* (wings removed) (*n* = 5)	right	5.3	4.98	5.81	6.08	5.95	6.12	
	left		5.53	6.63	6.79	6.76	6.82	
*Teleogryllus oceanicus* (wings removed) (*n* = 5)	right	4.6	4.79	5.81	6.05	5.97	5.82	
	left		4.62	5.85	6.32	6.21	6.21	

### 3.2. Changes in wing resonance

**Figure 4.**
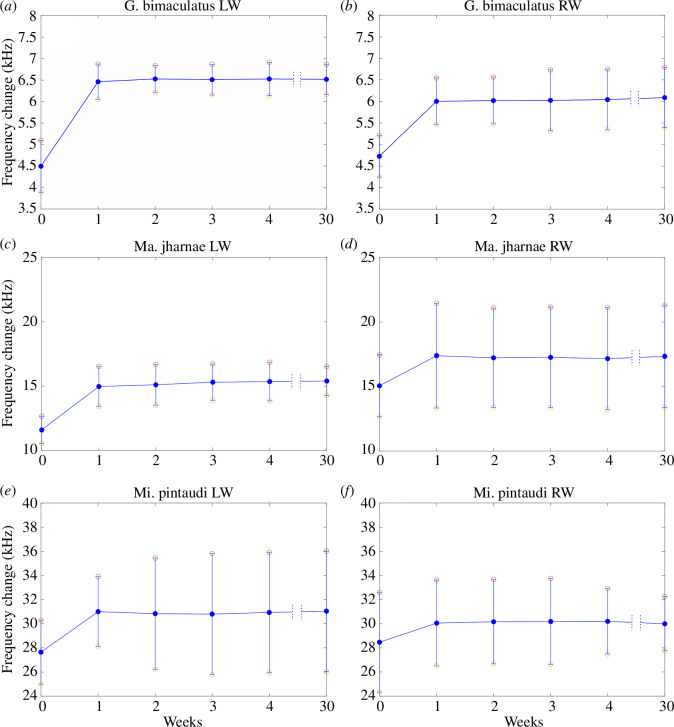
The average changes in wing resonance over a four-week period using laser Doppler vibrometry for attached wings. (*a, b*) The left and right wings of a male *G. bimaculatus*. (*c, d*) The left and right wings of male *Ma. jharnae*. (*e, f*) The left and right wings of male *Mi. pintaudi*. LW, left wing; RW, right wing.

Laser Doppler vibrometry scans on the attached forewings of *G. bimaculatus* reveal that, in life, the right wing has an average peak *f_o_* of 4.73 ± 0.48 kHz, while the left wing is slightly lower at 4.49 ± 0.61 kHz (table 1), supporting previous observations [14]. During the first week after death, there was a rapid increase in the *f*_o_. In the right wing, the *f*_o_ of 4.73 kHz increased to an average of 6.1 ± 0.54 kHz (figure 4*a*; table 1), while in the left *f*_o_ of 4.49 kHz increased to 6.46 ± 0.41 kHz (figure 4*b*; table 1). However, after this first week, there is very little change in the *f_o_* over the remaining weeks. The wings maintain a resonance between 5.96 and 6.1 kHz in the right (figure 4*a*) and between 6.46 and 6.53 kHz in the left (figure 4*b*).

A similar pattern can be seen in the wing resonances of *Ma. jharnae* and *Mi. pintaudi*. The right wing of *Ma. jharnae* had an initial average *f_o_* of 15.05 ± 2.4 kHz (table 1), while the left has a *f_o_* of 11.6 ± 1.05 kHz (table 1). During the first week, the right wing increased to 17.39 ± 4.06 kHz (figure 4*c*; table 1) and the left increased to 14.96 ± 1.56 kHz (figure 4*d*; table 1). These then plateaued and were maintained between 16.8 and 17.3 kHz in the right wing (figure 4*c*) and between 15.6 and 15.9 kHz in the left (figure 4*d*) for the remaining weeks.

In *Mi. pintaudi*, the right wing had an initial average *f_o_* of 28.46 ± 4.13 kHz (table 1), while in the left, it was 27.64 ± 2.64 kHz (table 1). In the first week, the right wing increased to 30.39 ± 4.25 kHz (figure 4*e*; table 1) and the left to 30.1 ± 4.25 kHz (figure 4*e*; table 1). These then plateaued and were maintained between 28 and 28.3 kHz in the right wing (figure 4*f*) and between 30.16 and 30.51 kHz in the left (figure 4*f*).

**Figure 5.**
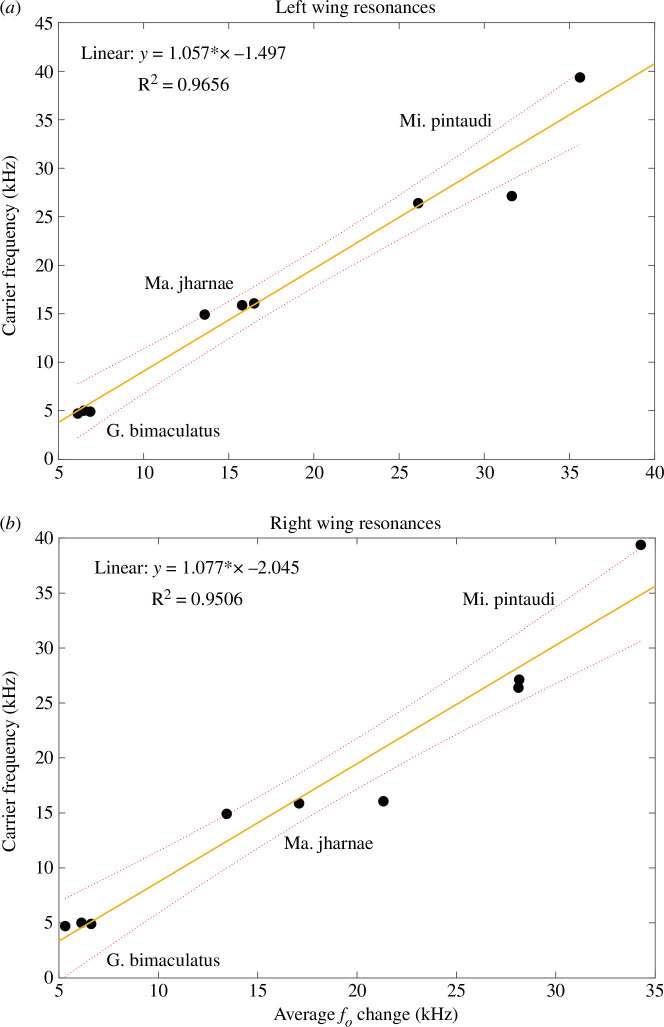
Average wing resonances displayed against calling song carrier frequency for attached wings. (*a*) The average resonances of the left wings plotted against carrier frequency. (*b*) The average resonances of the right wings plotted against carrier frequency. *f_o_*, resonant frequency.

For all individuals of the same species, we averaged the change in *f_o_* over the duration of the test. The right wings of *G. bimaculatus* had an average change in *f_o_* of 6.024 ± 0.044 kHz and 6.508 ± 0.024 kHz in the left wings. *Macrobinthus jharnae* had an average change in *f_o_* of 17.28 ± 0.079 kHz in the right wings and 15.287 ± 0.257 kHz in the left wings. Lastly, *Mi. pintaudi* had an average change in *f_o_* of 30.187 ± 0.499 kHz in the right wings and 31.121 ± 0.447 kHz in the left wings. These values were then plotted against the species *f_c_* providing a means to recover the original *f_c_* if the current *f_o_* is known (figure 5).

The removed wings of *G. bimaculatus* had an initial average resonance of 4.26 kHz in the right wing and 4.55 kHz in the left wing (table 1). This increased to an average of 5.14 kHz in the right wing and 5.41 kHz in the left before plateauing and was maintained for the remaining scans. The same trends can be seen in the removed wings of *G. assimilis* and *T. oceanicus* (see table 1).

### 3.3. Creating the model

The average change in *f_o_* was taken from each individual, and was obtained using LDV with wings attached and plotted against its *f_c_* (figure 5). A linear regression analysis was performed on this scan data, which revealed a positive relationship between the average change in *f_o_* and recorded *f_c_*, in both wings (figure 5). The left wings predict *f_c_* more accurately (*F* = 196, *p* = 2.23 × 10^−6^, s.e. = 0.075, d.f. = 7) (figure 5*a*) than the right wings (*F* = 135, *p* = 7.95 × 10^−6^, s.e. = 0.093, d.f. = 7) (figure 5*b*); however, both remain highly significant. Additionally, the *R*^2^ = 0.9656 (left wings) and *R*^2^ = 0.9506 (right wings) indicate that the models allow for the majority (>95%) of variance.

### 3.4. Wing resonance of long-preserved specimens

**Figure 6.**
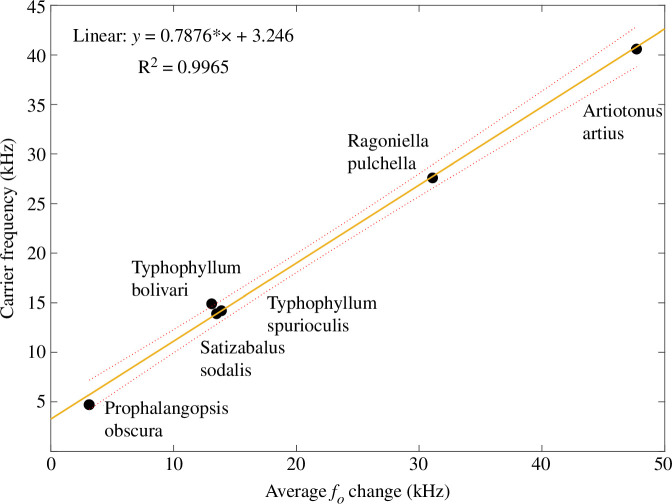
The recovered wing resonances of preserved specimens plotted with carrier frequency. *f_o_*, resonant frequency.

LDV scans on the forewings of preserved specimens reveal the effect 20+ years of desiccation have had on wing resonance. The *f_o_* of *Artiotonus artius* was scanned at 46.23 kHz, while *Prophalangopsis obscura* had a peak resonance of 4.8 kHz (table 2). *Ragoniella pulchella* (a species using broadband calls) returned a broadband response between 17.26 and 30.75 kHz and *Satizabalus sodalis* had a peak *f_o_* of 14.8 kHz. Lastly, *T. bolivari* and *T. spurioculis* had respective resonances at 14.1 and 14.484 kHz (table 2). Using the equation presented in figure 5, the scanned *f_o_* can be inputted to return the original *f_c_* (table 2). Recovered *f_c_* was then plotted against scanned *f_o_* revealing a positive relationship (*R*^2^ = 0.9965, *p* = 4.67 × 10^−6^) (figure 6).

**Table 2.** Calling song components of preserved specimens. *f_c_*, carrier frequency; *f_o_*, resonant frequency; FMZ, Fernando Montealegre-Z; LNHM, London Natural History Museum; *, broadband calling song.

species	preserved since	depository	scanned *f_o_* (kHz)	*f_c_* (kHz)	recovered *f_o_* (kHz)
*Artiotonus artius*	1995	FMZ private	46.23	40.6	47.7
*Prophalangopsis obscura*	1898–1939	LNHM	4.8	4.7	3.12
Ragoniella pulchella*	1997	FMZ private	17.26–30.75	27.6	16.5–31.1
*Satizabalus sodalis*	1997	FMZ private	14.8	14.2	13.9
*Typophyllum spurioculis*	2000	FMZ private	14.484	13.9	13.5
*Typophyllum bolivari*	2000	FMZ private	14.1	14.9	13.1

## 4. Discussion

In all the species used in this study, we show that the peak *f_c_* of the calling song is comparable to that of the *f_o_* of the wings (compare figures 1–3 with figure 4 and table 1), which is well known [4,5,10,14,16]. Through the use of LDV we confirm that *f_o_* increases over time as the wings desiccate. There were some fluctuations in resonance during recording, with the final resonance sometimes being lower than the third week of recording (table 1). This could be due to minor changes in humidity between recording sessions or because some wings dried in an awkward position. Wing structures and the amount of fluid content could all impact rates of desiccation. A future approach would be to attempt to rehydrate a desiccated wing and determine whether the living resonance can be recovered.

**Figure 7.**
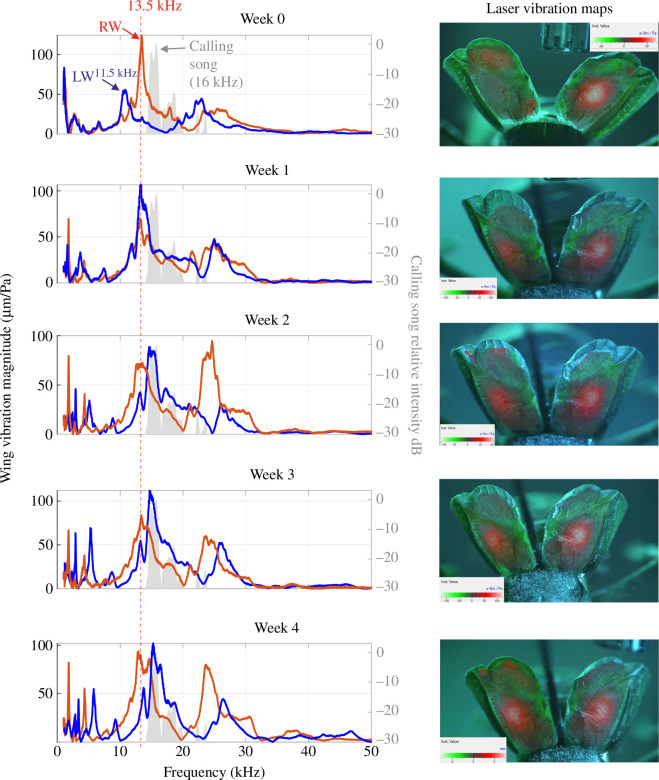
The change in wing resonance over a four-week period for the attached wings of a single male *Macrobinthus jharnae*. Power spectra comparing left (blue) and right (red) wing vibrations against calling song (grey) with images of the displayed wings. LW, left wing; RW, right wing.

For wings that remained attached to the body, the *f_o_* of both wings changed rapidly during the first week (figures 4 and 7). This increment is linear only during the first week, after which the resonance does not change and reaches a plateau in all species (figure 4). For wings that were removed from the body and attached to toothpicks, the initial *f_o_* was lower; however, there was still a rapid increase in both wings during the first week (table 1), which then levelled.

We noticed that the amount of change in *f_o_* is proportional to the *f_c_*. For example, for a 4.8 kHz calling song (the *f_c_* of *G. bimaculatus*), the average *f_o_* change is 7.6 kHz for the left wing (figure 5*a*) and 7.2 kHz (figure 5*b*) for the right wing. This leaves a difference of more than 2 kHz between the *f_c_* and the *f_o_*. This allows us to accurately recover the *f_c_* of dead and preserved specimens if the wings are intact and attached. Once the wings are scanned using LDV to obtain the *f_o_*, these values can be inputted into the models presented in figure 6, allowing the original *f_c_* to be recovered. We confirmed this by scanning the attached wings of several long-preserved insects. The *f_o_* of *T. bolivari* was 14.1 kHz (table 2), which when inputted into the model returns a *f_c_* of 13.1 kHz, comparable to its reported *f_c_* of 14.9 kHz [21] (figure 6). The *f_o_* of *T. spurioculis* was 14.484 kHz (table 2), which returns a *f_c_* of 13.5 kHz, similar to the reported *f_c_* of 13.9 kHz [22] (figure 6). The *f_o_* of the right mirror of *S. sodalis* was 14.8 kHz (table 2); once inputted into the model, the *f_c_* is returned as 13.9 kHz (figure 6), while the true *f_c_* of *S. sodalis* was recorded at 14.2 kHz [23]. The aforementioned species all produce a pure-tone call. *Ragoniella pulchella* produces a broadband call; therefore, inputting a single peak *f_o_* would not return the accurate *f_c_* (see §4.1). However, once the minimum and maximum peaks in the frequency spectrum (17.26–30.75 kHz) are inputted into the model, they return an accurate *f_c_* range of 16.5–31.1 kHz. Other examples of the model being used to recover the *f_c_* of preserved specimens can be seen in table 2.

This model (figure 5) has the potential to be applied to a variety of fields, the most obvious being preserved museum specimens. It offers a non-invasive method to recover the *f_c_* of long-dead specimens that may have never been recorded before, of which there are many [24], such as *Prophalangopsis obscura* [20]. This opens new research directions and projects using museums’ insect collections and can have a positive impact on biodiversity studies and species diversification using sound, for example, bridging the gaps produced by species for which the acoustic features are unknown. Finally, these studies might also give museums alternative routes to justify their biological collections. We do, however, foresee there being some limitations.

### 4.1. Limitations and advice

A major limitation is that an LDV is needed. Although in this research we used a micro-scanning LDV, a single-point LDV can provide a good assessment of resonance, reducing cost.

In order to avoid damaging preserved specimens, it is most ideal if they have been pinned with their wings spread apart allowing for easy scanning of the resonating areas. Otherwise, they would need to be relaxed to spread the wings.

While we show that carrier frequency can be accurately approximated from preserved specimens, LDV is not able to interpret the animal’s acoustic behaviour. This method is unable to recover other features of the calling song such as call duration or the structure of a syllable. The predication of *f_c_* also assumes that there is negligible variation in the other structures that serve an important purpose in sound production during stridulation.

The proposed method can be used reliably in species producing pure tones or calls with symmetrical spectra. For those with complex broadband spectra, the situation becomes more challenging as these normally involve resonators with several resonant peaks. It is possible to recover these individual peaks using the model, such as in *R. pulchella*; however, this should be studied in more detail.

## 5. Conclusions

Through the use of LDV we confirm that the *f_o_* of the Ensiferan forewings increases after death due to the process of desiccation. After the first week, there is a sharp increase in *f_o_* (figures 4 and 5; table 1). However, this increment is linear only during the first week, after which the resonance stabilizes and reaches a plateau (figure 4). Using the given data, we propose a model that can be used to recover the key song components of preserved Orthoptera (figure 5). We demonstrate the model’s effectiveness on readily available animals (figure 5) as well as on long-preserved specimens maintained in entomological collections (figure 2; table 2).

## Data Availability

All raw data can be accessed and downloaded via this reference: [25]. Supplementary material is available online [26].
